# Proteomic and metabolomic profiles demonstrate variation among free-living and symbiotic *vibrio fischeri* biofilms

**DOI:** 10.1186/s12866-015-0560-z

**Published:** 2015-10-23

**Authors:** Alba Chavez-Dozal, Clayton Gorman, Michele K. Nishiguchi

**Affiliations:** Department of Biology, New Mexico State University, Box 30001, MSC 3AF, Las Cruces, NM 88003-8001 USA

**Keywords:** *V. fischeri*, Symbiosis, Biofilms, Planktonic, Mass spectrometry, Liquid chromatography, Metabolomics, Proteomics

## Abstract

**Background:**

A number of bacterial species are capable of growing in various life history modes that enable their survival and persistence in both planktonic free-living stages as well as in biofilm communities. Mechanisms contributing to either planktonic cell or biofilm persistence and survival can be carefully delineated using multiple differential techniques (*e.g*., genomics and transcriptomics). In this study, we present both proteomic and metabolomic analyses of *Vibrio fischeri* biofilms, demonstrating the potential for combined differential studies for elucidating life-history switches important for establishing the mutualism through biofilm formation and host colonization.

**Methods:**

The study used a metabolomics/proteomics or “meta-proteomics” approach, referring to the combined protein and metabolic data analysis that bridges the gap between phenotypic changes (planktonic cell to biofilm formation) with genotypic changes (reflected in protein/metabolic profiles). Our methods used protein shotgun construction, followed by liquid chromatography coupled with mass spectrometry (LC-MS) detection and quantification for both free-living and biofilm forming *V. fischeri*.

**Results:**

We present a time-resolved picture of approximately 100 proteins (2D-PAGE and shotgun proteomics) and 200 metabolites that are present during the transition from planktonic growth to community biofilm formation. Proteins involved in stress response, DNA repair damage, and transport appeared to be highly expressed during the biofilm state. In addition, metabolites detected in biofilms correspond to components of the exopolysaccharide (EPS) matrix (sugars and glycerol-derived). Alterations in metabolic enzymes were paralleled by more pronounced changes in concentration of intermediates from the glycolysis pathway as well as several amino acids.

**Conclusions:**

This combined analysis of both types of information (proteins, metabolites) has provided a more complete picture of the biochemical processes of biofilm formation and what determines the switch between the two life history strategies. The reported findings have broad implications for *Vibrio* biofilm ecology, and mechanisms for successful survival in the host and environment.

**Electronic supplementary material:**

The online version of this article (doi:10.1186/s12866-015-0560-z) contains supplementary material, which is available to authorized users.

## Background

Among biofilm communities, there are multiple biochemical interactions that shape the dynamic community that contrasts from free-living planktonic cells. Specifically, the notable stress resistance of biofilms has been associated with physiological changes that bacteria undergo during the transition to the biofilm state [1]. A vast amount of research in the last decade has focused on characterizing unique aspects of microbial biofilms, which include genomics and post-genomic functional approaches. These techniques have allowed a comparative molecular characterization of bacterial communities during various life history stages [2–4]. Numerous techniques include 16S rRNA sequencing for community composition analysis [5], mutational analysis of particular genes, RNA profiling [6], genomics [7], transcriptomics for capturing global views of genetic diversity and expression, and isotope-probing to link phylogeny with community function and processes [8]. These pioneering studies were helpful for their initial differential analysis of biofilm life history strategies, and have opened the way to genetic characterization of biofilm-single cell transitions in phenotype.

New approaches have become available that allow a complete differential profile including proteomic and metabolomic analysis [9]. Proteomic profiles help dissect the complexity of microbial communities by analyzing protein expression, function, modification, and interactions over temporal scales. Specific separation techniques coupled with mass spectrometry (MS) analysis are also essential for proteomic profiling of these extensive and diverse populations. One classic approach is the combination of two-dimensional (2D) protein gel electrophoresis followed by spot identification via isoelectric focusing [10]. In addition, high-throughput approaches are available for protein profiling such as shotgun proteomics, where proteins are digested and the generated peptides are identified by capillary liquid chromatography in tandem with mass spectrometry [10].

Metabolomics refers to the analytical approach used to study different cell products (“chemical fingerprints”) that help to understand the physiological state of microorganisms [11]. This analysis is achieved with the use of liquid chromatography coupled with mass spectrometry (LC-MS), followed by detection, and quantification. Subsequent identification of metabolites is then accomplished by cluster analysis and mapping [11]. Although each type of analysis produces an extensive amount of information, combining complementary techniques could significantly contribute to our understanding of biofilm developmental processes. A more recent approach termed “meta-proteomics” aims to identify and combine protein and metabolic data to bridge the gap between phenotypic changes (planktonic cell to biofilm formation) with genotypic changes (reflected in protein/metabolic profiles) [12]. Earlier studies have integrated various system biology analyses (including proteomics, metabolomics and transcriptomics) for biofilms formed by *Bordetella pertussis* [13], *Leptospirillum sp.* [14], and *Pseudomonas fluorescens* [15]. However, there are no studies that integrate data collected via proteomic/metabolomic (meta-proteomics) data for *Vibrio* biofilms.

*Vibrio fischeri* is a mutualistic bioluminescent bacterium that infects the light organs of sepiolid squids and monocentrid fishes. *V. fischeri* produces bioluminescence that is used by the squid to avoid predation in a behavior known as counterillumination [16]. The mutualism is established when the host provides an appropriate niche for the bacteria to reproduce at much higher rates than in their free-living state [17]. *V. fischeri* is capable of forming biofilms both in seawater during its free-living stage and inside its host squid’s light organ while in symbiosis [18]. Environmental and mutualistic biofilms differ in the sense of bacterial diversity, where multispecies biofilms dominate the seawater environment [1] and only one or a few *Vibrio* bacteria colonize and form biofilm inside the squid’s light organ [19–22]. The ability of *V. fischeri* to form a biofilm community within its squid host plays a central role in the establishment and maintenance of the mutualism, as well as the degree to what functional molecules are produced and overexpressed for biofilm formation. Therefore, this study aims to describe the metabolic and proteomic profiles of a monospecies biofilm that is crucial for understanding what bacterial molecular components are important for establishing this mutualistic association. Proteomic and metabolomic analyses for both free-living (planktonic) and monospecies biofilm were completed for *V. fischeri* strain ETJB1H to provide the first partial *Vibrio* meta-proteome profile for single cell-to-biofilm physiology. Metabolomic analysis indicates several molecular changes that are the result of different biosynthetic pathways associated with exopolyssacharide (EPS) production and biofilm formation, as well as proteins that are important for persistence in the seawater environment.

## Methods

### Microorganism and biofilm formation experiments

*Vibrio fischeri* ETJB1H was isolated from the light organ of *Euprymna tasmanica* from Jervis Bay, Australia [23] and was used throughout this study. *V. fischeri* ETJB1H was routinely cultured on Luria Broth High Salt (LBS, 10 g tryptone, 5 g yeast extract, 20 g sodium chloride, 50 mL 1 M Tris pH 7.5, 3.75 mL 80 % glycerol and 950 mL distilled water) agar (15 %) and sub-cultured on LBS liquid media at 28 °C. Biofilm formation was grown as previously described [24, 25]. Briefly, three flasks of 250 mL with 100 mL of LBS media were inoculated with a 1:100 dilution from an overnight culture (of a 1.0 McFarland standard) and incubated for 24 h at 28 °C under static conditions. After incubation, planktonic cells were removed and flasks were briefly washed with LBS to remove any excess planktonic cells. Biofilm-forming bacteria (the ones that were tightly attached to the glass of the flask) were then removed by placing 100 mL of LBS and sonicating for 10 s three times under low intensity (40 %) power using the Bransonic 220 sonicator (Branson. Ultrasonic, Danbury, CT, USA). Biofilm cells were then concentrated and washed three times by spinning down the cultures at 10,000 xg for 15 min and removing any excess supernatant media. Each of the samples were divided in equal amounts for their proteomic 2-D page and shotgun analysis respectively (3 combined samples for the 2D page analysis and 3 combined samples for the shotgun analysis).

### Protein preparation and 2-D PAGE electrophoresis

For protein extraction, we used the EasyLyse™ bacterial protein extraction solution (Epicentre technologies, Madison, WI) following manufacturer’s instructions. In brief, a lysis solution was prepared as follows: 0.5 mL of D.I. water, 2 μl of 1 M MgCl_2_, and 0.5 mL of Lysis Buffer and 1 μl of enzyme were added. A cell pellet consisting of aproximatelly 10^9^ cells was added to 200 μL of the above solution. After incubation at room tempareture for 5 min, the samples were centrifugated and the supernatant (cell paste) was transferred to a clean tube. Fifty micrograms of each cell paste was prepared for first dimensional isoelectric focusing by adding four parts of lysis solution (7 M urea, 2 M thiourea, 1 % dithiothreitol, 2 % Pharmalyte 3–10, 0.5 % Triton X-100, 0.14 % phenylmethylsulfonyl fluoride) to one part of protein sample (volume per volume) as described previously [26].

The proteins are initially separated in the first dimension based on their isoelectric points; the focused proteins of the first dimension are subsequentelly separated in a second dimension based on their molecular masses. First, dimensional separation was completed using 17 cm IPG strips, pH 3–10. One microgram of the protein sample was loaded and isoelectric focusing was performed following the manufacturer’s protocol (Bio-Rad, Richmond, CA). PROTEAN© IEF cell was used for the first separation at settings of 150 kVh and 23 °C. Strips were then equilibrated for 15 min in a buffer containing 2 % SDS, 6 M urea, 0.05 M Tris–HCl, pH 8.5, and 20 % glycerol with 2 % DTT (Dithiothreitol) and equilibrated again in the same buffer with 2.5 % iodoacetamide. The equilibrated strips were transferred to a PROTEAN II© version xi cell tank for second dimension run (30 mA per gel) in 10 % polyacrylamide gel, and then visualized after staining with Coomasie brilliant blue R250. Stained cells were covered with cellophane and air-dried overnight at room temperature. Gels were analyzed pairwise by eye for differences in their protein patterns [26]. In addition, differential analysis by Guild BioSciences proteome analysis service and a computer densitometric analysis of spots were completed using the Image Master Platinum 5.0 software (GE Healthcare, PA). A threshold of 2-fold change was used to determine significance between biofilm and planktonic groups. Gels were analyzed pairwise by eye for differences in their protein patterns by overlaying the gels on a light table, gels were then scanned into a computer graphics program (Adobe Photoshop 5.0) and one replicated is used as a reference.

### Protein shotgun analysis

A fraction of the whole protein extractions (approximately 200 μL, which corresponds to 1 mg of total protein) were trypsin-digested. For digestion, the sample was reduced by adding 5 μL of DDT (Dithiothreitol, 200 mM: 1 mL of 100 M NH_4_HCO_3_ and 30.86 mg of DTT) and boiled for 10 min and then incubated for 1 h. Alkylation was achieved by adding 4 μL of iodoacetamide (1 M: 200 μL of 100 mM NH_4_HCO_3_ and 37 mg of iodoacetamide) and incubated for 1 h. Neutralization of the remaining iodoacetamide was achieved by adding 20 μL of DTT and incubating for 1 h. Trypsin was added to the mixture (1 mg for every 50 mg of protein) and complete digestion was accomplished after incubating for 18 h at 37 °C. Protein digests (approximately 100 μM) were analyzed by tandem mass spectrometry through cation exchange-reversed phase chromatography, utilizing a hybrid linear ion trap FT-ICR mass spectrometer with ultra performance liquid chromatograph (UPLC/MS, Agilent Technologies 110 Series, CA) with a capillary system attached to a quadruple ion time (Thermo LQT, Thermo Fisher Scientific, CA). Three technical replicates were analyzed for each combined sample. Peptide libraries were collected in a database (as a single .mgf file for each sample) searched against a marged database composed of reviewed entries of Uniprot database and analyzed with Mascot search engine (www.matrixscience.com). Mascot parameters include proteolysis by trypsin/chymotrypsin with size tolerances of 0.5 Da for peptide fragments, with a 95 % probability that the protein identified is not a random match. The false discovery rate (FDR) was calculated using the automated decoy database tool in MASCOT where decoy statistics were automatically calculated for all matches. Alternatively, FASTA sequences of target peptides previously identified were run in the program peptide cutter (web.expasy.org/peptide_cutter) and resulting fragments were compared to those identified in our analysis. A score of 35 % matching peptides (or higher) indicates a protein match [27].

### Metabolomic analysis

To prepare samples for metabolite extraction, strains were inoculated in triplicate in 120 mL of Luria Bertani high salt media (LBS; per litre composition: 10 g tryptone, 5 g yeast extract, 20 g NaCl, 50 ml 1 M tris pH 7.5, 3.75 ml 80 % glycerol and 950 mL dH_2_O). When cultures reached an OD_580_ of 1.0, they were pelleted at 10,000 xg for 10 min at 4 °C. Pellets were re-suspended in 10 mL of ice-cold phosphate buffered saline (PBS, pH 7.4) and cells were pelleted again under the same conditions. The supernatant was discarded and pellets were snap-frozen with liquid nitrogen. Bacterial cells were lyophilized for 24 h (Labconco model 7740020) and were analyzed by the Biotechnology Center at the University of Illinois at Urbana-Champaign (Metabolomics Center, Roy J. Carver Biotechnology Center, University of Illinois at Urbana-Champaign). The approach used was a two-step LC/MS (Applied Biosystems 5500 QTrap with Agilent 1200 LC, Agilent Technologies, CA and Applied Biosystems CA) followed by targeted identification of differentially expressed metabolites using quadrupole time of flight (Q-TOF) MS/MS. Three technical replicates were used for this analysis.

## Results

*V. fischeri* cells were grown in parallel as the planktonic (free-swimming) culture and as biofilms on the glass surface of the flask to identify and compare differences in protein profiles from the two physiological states. Cell viability was not affected after sonication for the biofilm cells, and there was no statistical difference between the number of Colony Forming Units of planktonic and biofilm samples collected after incubation time (24 h at 28 °C) for an OD_600_ = 1.0 (data not shown). The profiles presented correspond to a mature biofilm (structure achieved between 18 and 24 h of incubation) and free-living cells that did not form any biofilm. Two proteomic analysis approaches were used including i) Complete protein profile obtained by spot analysis, followed by differential two-dimensional gel electrophoresis, and ii) trypsin protein fractionation followed by shotgun identification (UPLC/MS) and peptide analysis (Mascot, peptide cutter). Metabolomic analysis was achieved through liquid chromatography coupled with mass spectrometry (LC/MS).

### Identification of biofilm protein fractions by two-dimensional gel electrophoresis

**Fig. 1:**
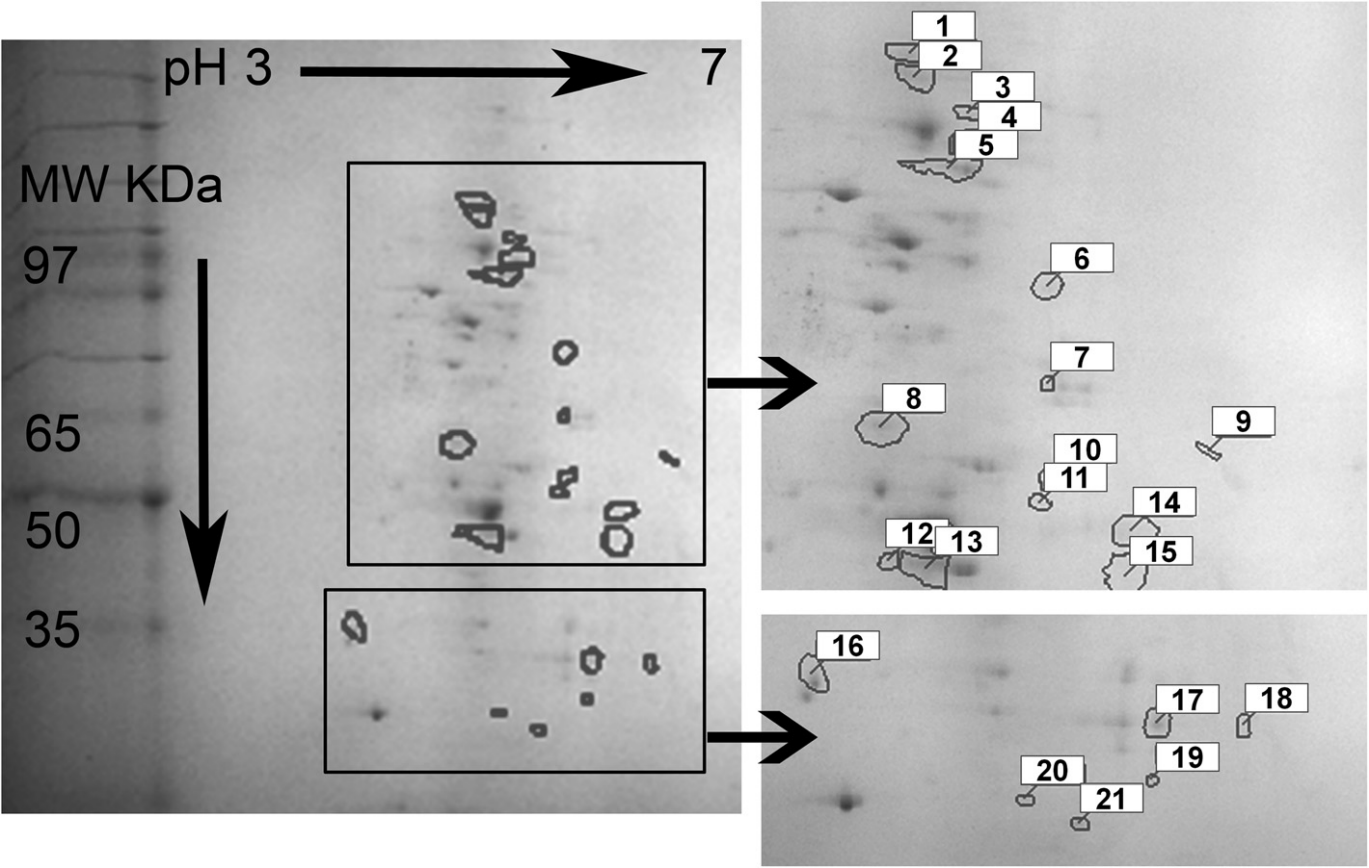
2D-PAGE gel of the up-regulated *Vibrio fischeri* ETJB1H biofilm protein exudate. Colored squares correspond to magnification of fractions of the gel indicating a better resolution of spots. Numbers identify up-regulated proteins described in Table 1

**Fig. 2:**
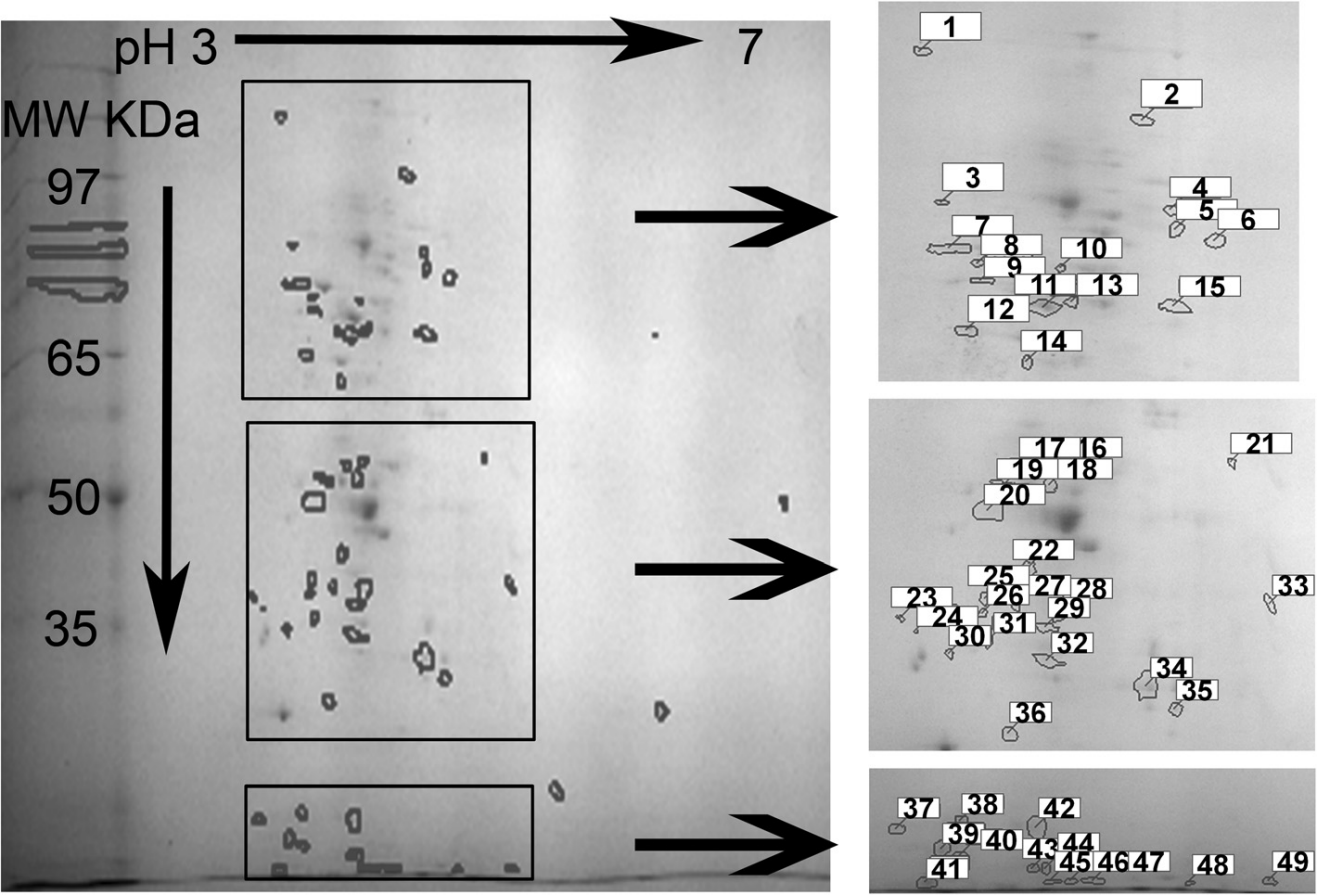
2D-PAGE gel of unique *Vibrio fischeri* ETJB1H biofilm protein exudates. Colored squares correspond to magnification of fractions of the gel indicating an increased resolution of spots. Numbers identify unique biofilm proteins described on Table 2

Patterns of protein expression in biofilm communities were complex with an average of approximately 300 spots per gel. Using the planktonic protein gel as the reference, protein spots detected in the biofilm profile were matched against the reference. The number of matched spots was 140 with a total of 75 % gel coverage. Of these, 21 spots were up-regulated in a magnitude of 2 fold or more (Fig. 1 and Additional file 1: Figure S1). Table 1 lists the assay parameters used (isoelectric point and molecular weight of proteins) that correspond to spots numbered in Fig. 1. In addition, there were 59 spots that were unique to the biofilm state (not present in the planktonic stage) that are indicated in Fig. 2 and Table 2. All the proteins listed from this analysis could be detected reproducibly in the range of 15 to 130 KDa.

**Table 1:** Biochemical properties of proteins identified to be up-regulated in the biofilm state of *Vibrio fischeri* ETJB1H

Spot	Isolectric point	Molecular weight (KDa)
1	3.97	111.51
2	3.98	105.53
3	4.20	96.30
4	4.22	89.63
5	4.12	84.35
6	4.52	70.82
7	4.52	59.62
8	3.85	55.37
9	5.17	53.52
10	4.53	51.82
11	4.49	50.35
12	3.88	47.64
13	4.03	47.27
14	4.86	49.00
15	4.83	46.98
16	3.22	39.60
17	4.68	35.06
18	5.05	34.74
19	4.65	29.76
20	4.12	28.51
21	4.35	30.66

Spot numbers correspond to proteins labeled in Fig. 2

**Table 2:** Biochemical properties of proteins identified to be unique in the biofilm state of *Vibrio fischeri* ETJB1H

Spot	Isolectric point	Molecular weight (KDa)
1	3.33	164.33
2	4.37	121.17
3	3.43	92.69
4	4.51	90.38
5	4.53	85.01
6	4.72	82.48
7	3.47	80.78
8	3.60	78.15
9	3.62	75.72
10	3.99	77.34
11	3.91	72.59
12	3.55	70.00
13	4.04	73.30
14	3.83	65.34
15	4.52	72.74
16	4.01	53.04
17	3.87	52.69
18	3.96	51.41
19	3.7	51.41
20	3.61	49.57
21	5.00	53.39
22	3.84	46.03
23	3.10	42.68
24	3.19	41.60
25	3.59	44.19
26	3.58	43.08
27	3.76	43.74
28	4.02	43.37
29	3.94	41.94
30	3.38	39.32
31	3.61	40.52
32	3.95	38.46
33	5.22	43.83
34	4.52	35.37
35	4.68	32.56
36	3.73	29.77
37	3.16	33.45
38	3.52	32.81
39	3.40	31.40
40	3.52	28.54
41	3.32	15.39
42	3.93	33.38
43	3.91	20.75
44	3.98	20.75
45	4.02	15.77
46	4.13	15.77
47	4.25	15.77
48	4.78	15.39
49	5.22	15.77

### Tandem mass spectrometry of peptides

The first scale proteomic analysis for *V. fischeri* using two-dimensional electrophoresis provides us with differentially expressed protein profiles that include both isoelectric points and molecular weight. In order to identify proteins that were present in the biofilm state of *V. fischeri,* a shotgun approach was used to detect differentially expressed proteins by matching peptide mass data to available proteome sequence databases (www.ncbi.nlm.nih.gov and www.uniprot.org) using the keyword *“Vibrio fischeri* ES114”. Additional analyses utilized the Mascot database. In order for a protein to be identified and considered present, tryptic peptides were required to be the primary identified hit in the database, and digests had to match at least 35 % of the complete protein compared with NCBI and Uniprot databases. In addition, theoretical isolectric points for the protein matches were calculated using algorithms from AnTheProt (antheprot-pbil.ibcp.fr) and Scansite3 (scansite3.mit.edu/#home). The criteria applied for identification resulted in a list of peptides that correlate with the molecular weight and isoelectric point detected for some protein spots observed to be either unique or up-regulated during biofilm production (Table 3).

**Table 3:** Summary of *Vibrio fischeri* ETJB1H biofilm proteins identified by UPLC/MS

Reference^a^	Protein identified	Theoretical MW(KDa)/IP^b^	No. peptides matched	Coverage (%)
GI:59711008	UDP-N acetylglucosomine 1 carboxyvinyltransferase	44.72/5.28	20	60
GI:59479183	Outer membrane protein U porin OmpU	33.18/3.92	12	55
GI:59712357	Cob(I)yrinic acid a,c-diamide adenosyltransferase	28.31/4.08	10	53
GI:59710825	ABC transporter ATP-binding protein	72.70/4.50	22	48
GI:197317623	carbon starvation protein A	53.51/5.14	13	44
GI:59712372	ATP-dependent Clp protease ATP-binding subunit	82.58/4.8	31	40
GI:31414756	sigma 54 transcriptional activator	53.89/5.15	12	37
GI:59482580	multidrug efflux system protein	122.42/4.87	34	37
GI:59480318	phosphate-binding protein	29.45/5.00	8	36
GI:59710693	Oxidoreductase	43.69/5.25	10	36
GI:121308572	Bioluminescence regulatory protein	84.37/4.20	24	35
GI:172087731	Flagellin	48.09/4.05	9	35
GI:59711558	putative Holiday junction DNA helicase RuvA	29.78/4.71	9	35
GI:148536406	RecA protein	28.54/4.32	8	35
GI:59480175	chaperone, DnaK-like protein	69.88/4.52	10	35

^a^NCBI reference sequence

^b^Calculated using AnTheProt (http://antheprot-pbil.ibcp.fr/) and Scansite3 (http://scansite3.mit.edu/#home)

Proteins related to multiple cellular processes were identified and listed in Table 3. The highest match corresponds to the catalytic enzyme UDP-N acetylglucosomine 1 carboxyvinyltransferase (UDP-GlcNAc) with a calculated MW/IP match to spot 33 (Table 2). Another enzyme detected corresponds to the adenosyltransferase cob(I)yrinic acid a,c-diamide transferase (MW/IP match in #20 of Table 1). Porins and membrane transporters were detected in *V. fischeri* ETJB1H biofilms, including outer membrane protein U (OmpU; #37 in Table 2), membrane transporter ABC (#11 in Table 2), and multidrug efflux pump (#2 in Table 2).

Interestingly, multiple stress-related proteins were detected in our study. These include the heat-stress response-related ATPase Clp protease (#6, Table 2), the carbon starvation protein A (#9, Table 1), the specific helicase RuvA related to Holiday junction formation (# 19, Table 1), the DNA double-strand repair protein RecA (#19, Table 1), the chaperone DnaK (#6, Table 1) and the transcriptional activator sigma 54 (#9, Table 1). Another set of proteins include flagellin (#13, Table 1), bioluminescence regulator (Lux R; #5, Table 1), oxidoreductase (#33, Table 1) and phosphate binding protein (#19, Table 1).

### Metabolomic analysis of *Vibrio fischeri *ETJB1H biofilms

A mass spectrometry-based profiling method was used for constructing the metabolome of *V. fischeri* ETJB1H planktonic and biofilm stages. This comparison is the first metabolomic study to determine the chemical fingerprint of *V. fischeri* biofilms, as well as the important biochemical pathways involved in formation from planktonic state to the mature biofilm of *V. fischeri*.

**Fig. 3:**
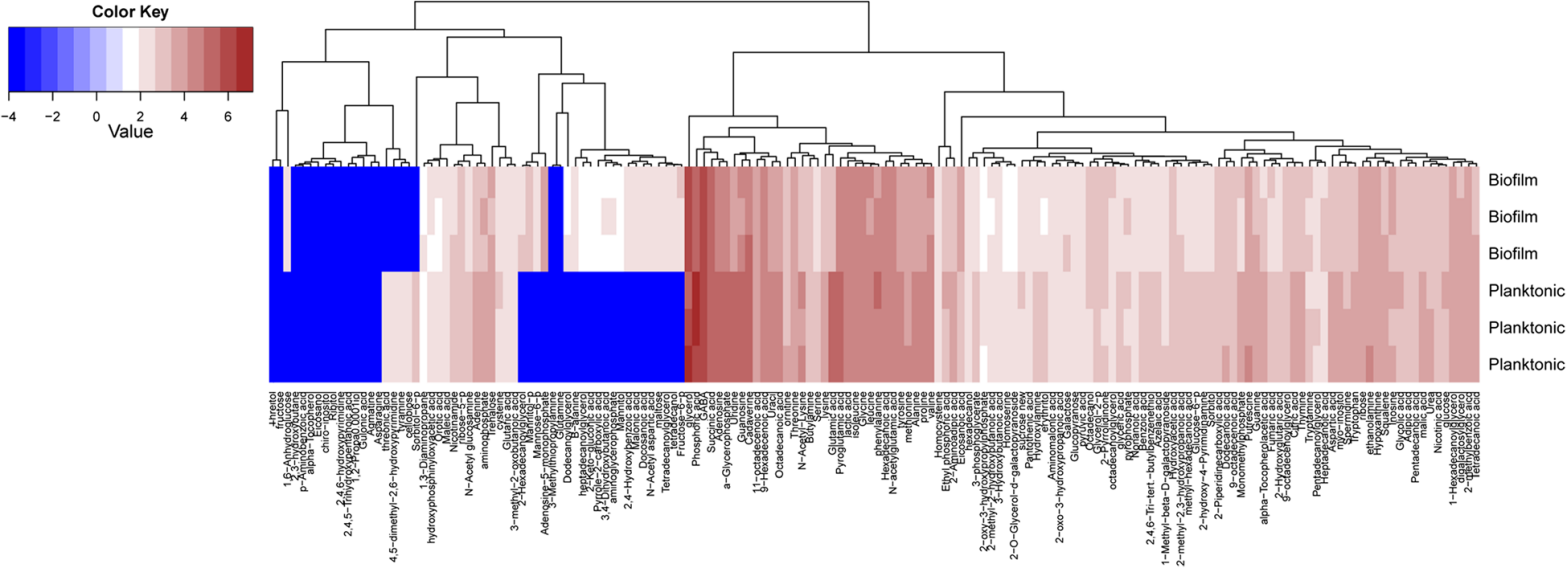
Heat map indicating the metabolomic profile of biofilm and planktonic ETJB1H *Vibrio fischeri* strains. Data are shown in triplicate as a colored map reflecting the logarithms that relate to the metabolite changes (red areas indicate an increase in metabolite abundancy, blue areas depict a decrease). Log values are color coded as indicated in the scale

Nominated altered chemicals (or biomolecules) were identified in both the planktonic and biofilm samples and are listed in Fig. 3 in a form of a heat map with a subset of color-coded metabolites (around 200) indicating critical fold changes between the two states. A concordance analysis of the two metabolic signatures (corresponding to the average of three analysis per condition, biofilm versus planktonic) indicates significant differences in metabolite profiles. Results for biofilm signatures indicate, up-regulated differences (2 fold) detected for multiple organic acids including carboxylic, phosphoric, aspartic, docosanoic, malonic, hydrobenzoic and keto-gluconic, as well as important sugars such as fructose, mannose, and maltose. Glycerol-derived components were also detected (hexadecanoglycerol, dodecanoglycerol, heptadecanoglycerol, and tetradecanoglycerol), and alcohols included mannitol and tetradecanol. Components that were observed to be significantly down-regulated (2–3 fold) in biofilms and up-regulated in the planktonic state include threoic acid, hydroxypyrimidine, tyramine, and cellobiose (Fig. 3).

## Discussion

In this study, we selected a meta-proteomics approach in order to resolve functional differences between the two life-history stages for *V. fischeri* (biofilms versus planktonic cells), and to describe factors that are important for successful colonization of the host, since the biofilm studied here resembles the community found in sepiolid squid light organs (for example, monospecificity, enriched environment, and availability of carbon sources), *Vibrio fischeri* biofilms and planktonic cells have been observed previously under Scanning Electron Microscopy [28–31]. There are distinct morphological differences; for example, planktonic cells are observed with flagellum and different shapes of fimbriae [28], whereas biofilms are observed as a flocculent bacterial mass encapsulated in a polysaccharide matrix [29, 31] structure that is similar to the one observed in biofilms formed in the squid’s light organ [31].

Results indicate differences in protein expression levels, including the number of unique proteins detected, as well as those with enhanced levels of expression (Table 3). The two-dimensional approach used was limited to the inability to identify all the spots through mass spectrometry as previously reported [32]. Alternatively, identification of some proteins were achieved through shotgun proteomics (a combination of ultra-performance liquid chromatography and mass spectrometry or UPLC/MS), and allowed the comparison of both theoretical molecular weight and isoelectric points to experimental values from proteins identified in the two dimensional gel, which validated the combinational methodology. Proteins identified in this manner were then classified depending on their functions including: a) stress-response regulators, b) catalytic enzymes, c) transporters, d) metabolic enzymes, and e) structural proteins. Proteins that were previously reported overexpressed include those involved in energy generation (*e.g*., succinyl-CoA synthase) and in biosynthesis (*e.g*., ribosomal factors) [33–43]. Future studies will focus on more specific differences between these two community phenotypes (environmental versus host biofilms), since it has been observed that environmental versus symbiotic strains respond differently to stress, including fluctuations in temperature and salinity [25].

### Upregulated proteins are important for maintenance and integrity of *V. fischeri* biofilms

In *Vibrio fischeri,* flagellin is expressed constitutively, and is esseantial for host colonization [39–41]. *V. fischeri* contains between one and five flagellar filaments that form a tuft of polar sheathed flagella [39]. *V. fischeri* flagellins are more similar to each other than to flagellins of other *Vibrio* species [38, 39], and therefore was not possible to differentiate the type of flagellin detected in this study. However, two proteins detected might be the closest match: (i) the flagellin FlgA that has been reported to be important for initial stages of host colonization [39] and (ii) FlgF, found to be important for host colonization and biofilm formation [39, 41].

Stress response proteins were also identified in this study. Elevated expression of the chaperone Clp was detected here. Clp proteins are known to regulate virulence in pathogenic bacteria such as *Porphyromonas gingivalis* [42] and *Vibrio cholerae* [43], and increased concentrations observed in *V. fischeri* biofilms might be related to increased success in host colonization, since it is believed that strong biofilm formers are also excellent host colonizers [1]. More interestingly, as reported for *V. cholerae*, Clp chaperone may be linked to the control of oxidative stress within the biofilm matrix (this would also include oxidoreductase, which was detected in this study). Oxidative stress is thought to be a result of a combination of slow growth in conjunction with a shift in oxygen at different depths of the biofilm [44]. Additional stress-related proteins were overexpressed in biofilms, including DnaK (molecular chaperone important for protein protection from denaturalization) [45] and the carbon starvation protein A (promotes peptide utilization during carbon starvation) [46]. It has been suggested that different micro-niches within the biofilm community are continuously exposed to various environmental stresses, inducing an increase of stress resistance mechanisms [47, 48].

Stress-inducible biofilm formation also produces DNA damage, which can trigger the bacterial SOS response initiated by the sensor protein RecA [49, 50], which was overexpressed in the biofilm samples. In addition, these methods detected the protein RuvA (responsible for Holiday junction formation as well as initiation of the SOS response). RuvA along with RuvB in the presence of ATP release the cruciform structure formed during strand exchange during homologous recombination [51], which might also occur in *Vibrio* biofilm communities. Since expression of proteins related to DNA repair can be synthesized up to 10 times more in biofilms [52, 53], this may result in undetectable traces in planktonic bacteria.

Another important component detected in *V. fischeri* biofilms is sigma factor 54 (σ^54^) that has been reported to be an important regulator of a wide range of bacterial processes, including nitrogen metabolism in *Escherichia coli* [54], biogenesis of flagella in *Vibrio parahaemolyticus* and *Vibrio cholerae* [55, 56], and bioluminescence in *Vibrio harveyi* [57]. More interestingly, σ^54^ in *V. fischeri* is encoded by the *rpoN* gene [58] that is overexpressed during the biofilm state, controlling flagellar biosynthesis (motility), nitrogen assimilation, luminescence, and biofilm formation [58].

Biofilms cells expressed a presumptive ABC transporter, which corresponds to a major class of translocation machinery in multiple bacterial species [59, 60]. ABC transporters have been previously identified to be differentially expressed during biofilm formation in *Pseudomonas aeruginosa* and *E. coli* [61, 62] and they may be linked to the transport of small molecules and solutes during the formation and maintenance of the mature biofilm compared to the cells in their planktonic state. In addition, the detected ABC transporter might influence cytoplasmic pH homeostasis by increasing transmembrane fluctuation of ions (for example K+) to allow compensation after pH stress (or osmoprotection). It is known that the light organ of sepiolid squids undergoes anaerobic stress based on fermentation genes expressed solely in the light organ environment [16], and possible drops in pH may be due to the acid by-products accumulating during this time.

The outer-membrane protein detected in this study (OmpU) has been previously identified in symbiotic *V. fischeri*, and has an important role in the initiation of colonization of the squid light organ [37]. In addition, disruption of the *ompU* gene results in increased sensitivity to membrane-disrupting chemical agents such as chlorine and organic acids [37]. These observations indicate that OmpU might have an important role in maintaining membrane integrity during *V. fischeri* biofilm development by providing defense mechanisms that are essential for resistance to the acidic environment within the biofilm matrix.

Biofilm development is guided by several regulatory systems. One of the important processes is formation of the exopolyssacharide (EPS) matrix, a hallmark of bacterial biofilms. UDP-GlcNAc (UDP-N acetylglucosomine 1 carboxyvinyltransferase) was detected in this study, and has an important role in the synthesis of EPS by acting as a transcriptional regulator [63].

Phosphatase-binding proteins have been described to increase production of the second messenger cyclic diguanylic acid (c-di-GMP) in *Pseudomonas aeruginosa* [64] and this protein was prevalent in our study. C-di-GMP is a central regulator of the prokaryote biofilm lifestyle [65], including *V. fischeri* biofilms.

There are multiple proteins that regulate bioluminescence. In particular, biofilm formation and bioluminescence are linked through proteins that regulate bacterial communication or quorum sensing [66]. One protein detected in this study (bioluminescence regulatory protein) may up-regulate the quorum sensing cascade, which, among other functions, has been reported to increase production of EPS [66]. This finding is particularly important for host-related biofilms and host survival since bacterial bioluminescence (increased in high bacterial density, as in the case of biofilms and not planktonic cells) is the main process that sepiolid squids use for the counterillumination (silhouette reduction from the moonlight at night). Therefore, in *V. fischeri*, regulation of bioluminescence is activated when bacterial concentration significantly increases in number and proximity (in the case of biofilms) [66–68]. Provided that the bioluminescence protein was detected in a biofilm that was formed under laboratory conditions, a similar bacterial community (and protein expression) is present in the squid host.

### Metabolomic profile revelas an increased number of components of the biofilm matrix

Most *Vibrio* biofilm matrices are composed of polysaccharides, such as the VPS (*Vibrio* polysaccharide), present in *V. cholerae* biofilms [1]. This metabolomic study detected carbohydrates that were present during the biofilm state and absent in planktonic cells. Proteins present consisted of mannose, maltose, fructose, and other monomeric sugars (galactose and glucose). In addition, smaller amounts of N-acetylglucosamine and N-acetyl glutamic acid were detected and have been described to be part of the VPS [69]. The presence of multiple glycerol-derived metabolites suggests that biofilm cells may use phospholipids released from neighboring cells, which possibly serve as a carbon source for amino acid biosynthesis. This metabolomic study revealed the presence of highly phosphorylated (and non-phosphorylated) glucans, which have been identified to be associated with the matrix of strains of *P. aeruginosa* [70]*.* Metabolites detected are important for synthesis of EPS components, or are related to regulatory processes involving second messengers (such as c-di-GMP). These components are strictly unique of community formation, but further research is required in order to determine if these metabolites are also important for mutualistic associations and what could be the metabolic differences between environmental and mutualistic biofilms. Future studies are needed in order to test whether glycerol and phosphate-derived components (detected in this study) are dominant in the squid’s light organ and how these components may contribute to host specificity and maintenance of symbiosis integrity.

## Conclusions

The objective of this study was to examine conserved proteomic and metabolomic signatures of both planktonic/free-living *V. fischeri* and their biofilm communities. Our results establish the methodology to utilize meta-proteomic analysis, enabling a more detailed perspective for understanding the biochemistry and metabolism of growth between the free-living/planktonic and community biofilm stages in a mutualistic bacterium. This meta-proteomic approach also improves the understanding of biofilms at a molecular level that is different from a transcriptomic or genomic comparisons (at the “functionality” level, which includes endpoint products, proteins, and metabolites). Results indicate a clear divergence associated with the restructuring of regulatory networks that allow community formation. Unique proteins and metabolites (mostly related to stress-responses, formation of the biofilm matrix and phosphorylated components) were significantly overexpressed in the biofilm state when compare to the free-living planktonic cells. Future work will entail combination of more differential studies (transcriptomics) to link the role of candidate genes to biochemical pathways and protein functionality.

## Additional file

Additional file 1: Figure S1. 2D-PAGE gel of unique spots presents in protein exudates from A) *Vibrio fischeri* ETJB1H planktonic cells and B) *Vibrio fischeri* ETJB1H biofilm cells. Circles indicate the spots that are unique for each protein extraction. Spot detection revealed 271 spots for the planktonic cells and 199 spots for biofilm cells. Using planktonic cells as the reference profile, there were a total of 21 spots upregulated and 52 downregulated for the biofilm cells. (DOCX 656 kb)

## Abbreviations

*V. fischeri*: *Vibrio fischeri*
ES114: *Euprymna scolopes* st. 114
ETJB1H: *Euprymna tasmanica* st. Jervis Bay 1H
2D-PAGE: Two-dimensional PolyAcrylamide Gel Electrophoresis
EPS: Exopolysaccharide
MS: Mass Spectrometry
LC: Liquid Chromatography
LBS: Luria Broth high Salt
IPG: Immobilized pH Gradient
mA: Milliampere
DTT: Dithiothreitol
SDS: Sodium Dodecyl Sulfate
FT-ICR: Fourier Transform-Ion Cyclotron Resonance
Da: Dalton
KDa: Kilodalton
FDR: False Discovery Rate
Q-TOF: Quadruple-Time of Flight
UPLC: UltraPerformance Liquid Chromatography
MW: Molecular Weight
IP: Isoelectric Point
c-di-GMP: Cyclic diguanylate
VPS: *Vibrio* Polysaccharide
