# Supplementary material for: Proteomic and metabolomic profiles demonstrate variation among free-living and symbiotic vibrio fischeri biofilms
Source: BMC Microbiol. 2015 Oct 23;15:226. doi: 10.1186/s12866-015-0560-z (PMC4619220; doi:10.1186/s12866-015-0560-z)
Supplement: Additional file 1: Figure S1. — 2D-PAGE gel of unique spots presents in protein exudates from A) Vibrio fischeri ETJB1H planktonic cells and B) Vibrio fischeri ETJB1H biofilm cells. Circles indicate the spots that are unique for each protein extraction. Spot detection revealed 271 spots for the planktonic cells and 199 spots for biofilm cells. Using planktonic cells as the reference profile, there were a total of 21 spots upregulated and 52 downregulated for the biofilm cells. (DOCX 656 kb) [file 12866_2015_560_MOESM1_ESM.docx]

**Supplementary material**

2

3
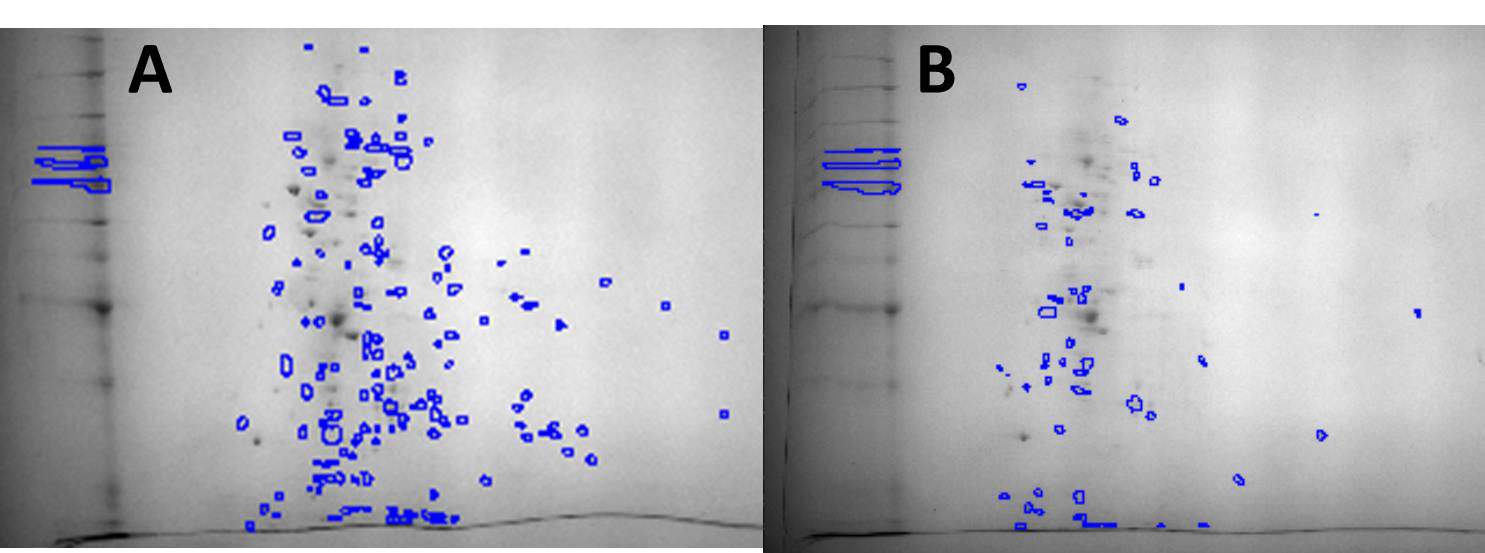


4

5

6 **Supplementary Figure 1.** 2D-PAGE gel of unique spots presents in protein exudates from A)

7 *Vibrio fischeri* ETJB1H planktonic cells and B) *Vibrio fischeri* ETJB1H biofilm cells. Circles

8 indicate the spots that are unique for each protein extraction. Spot detection revealed 271 spots

9 for the planktonic cells and 199 spots for biofilm cells. Using planktonic cells as the reference

10 profile, there were a total of 21 spots upregulated and 52 downregulated for the biofilm cells.

11

12

13

14
